# Association of ventilator-free days with respiratory physiotherapy in critically ill patients with Coronavirus Disease 2019 (COVID-19) during the first pandemic wave. A propensity score-weighted analysis

**DOI:** 10.3389/fmed.2022.994900

**Published:** 2022-09-12

**Authors:** Emilia Privitera, Simone Gambazza, Veronica Rossi, Martina Santambrogio, Filippo Binda, Davide Tarello, Salvatore Caiffa, Valentina Turrin, Carolina Casagrande, Denise Battaglini, Mauro Panigada, Roberto Fumagalli, Paolo Pelosi, Giacomo Grasselli

**Affiliations:** ^1^Healthcare Professions Department, Fondazione IRCCS Ca' Granda Ospedale Maggiore Policlinico, Milan, Italy; ^2^Thoracic Surgery and Lung Transplant Unit, Fondazione IRCCS Ca' Granda Ospedale Maggiore Policlinico, Milan, Italy; ^3^Internal Medicine Department, Respiratory Unit and Adult Cystic Fibrosis Center, Fondazione IRCCS Ca' Granda Ospedale Maggiore Policlinico, Milan, Italy; ^4^Respiratory Physiotherapy Equipe, ASST Grande Ospedale Metropolitano Niguarda, Milan, Italy; ^5^Intensive Care Respiratory Physiotherapy, Rehabilitation and Functional Education, San Martino Policlinico Hospital, IRCCS for Oncology and Neurosciences, Genoa, Italy; ^6^Rehabilitation Department, Santa Chiara Hospital, APSS di Trento, Trento, Italy; ^7^Anesthesia and Intensive Care, San Martino Policlinico Hospital, IRCCS for Oncology and Neurosciences, Genoa, Italy; ^8^Department of Medicine, University of Barcelona, Barcelona, Spain; ^9^Department of Anaesthesiology, Intensive Care and Emergency, Fondazione IRCCS Ca' Granda Ospedale Maggiore Policlinico, Milan, Italy; ^10^Department of Anesthesia and Intensive Care Medicine, ASST Grande Ospedale Metropolitano Niguarda, Milan, Italy; ^11^Department of Surgical Sciences and Integrated Diagnostics, University of Genoa, Genoa, Italy; ^12^Department of Pathophysiology and Transplantation, University of Milan, Milan, Italy

**Keywords:** respiratory physiotherapy, Intensive Care Unit, ventilator-free days, COVID-19, critical illness

## Abstract

**Background:**

Respiratory physiotherapy is reported as safe and feasible in mechanically ventilated patients with severe Coronavirus Disease (COVID-19) admitted to Intensive Care Unit (ICU), but the short-term benefits remain unclear.

**Methods:**

We performed a retrospective observational study in four ICUs in Northern Italy. All patients with COVID-19 admitted to ICU and under invasive mechanical ventilation (MV) between March 1^st^ and May 30^th^, 2020, were enrolled into the study. Overlap weighting based on the propensity score was used to adjust for confounding in the comparison of patients who had or had not been treated by physiotherapists. The primary outcome was the number of days alive and ventilator-free (VFDs). The secondary outcomes were arterial partial pressure of oxygen (PaO_2_)/fraction of inspired oxygen (FiO_2_) ratio (P/F) at ICU discharge, ICU length of stay, ICU and hospital mortality, and survival at 90 days. The trial protocol was registered on clinicaltrials.gov (NCT 05067907).

**Results:**

A total of 317 patients were included in the analysis. The median VFDs was 18 days [interquartile range (IQR) 10; 24] in patients performing physiotherapy and 21 days (IQR 0; 26) in the group without physiotherapy [incidence rate ratio (IRR) 0.86, 95% confidence interval (CI): 0.78; 0.95]. The chance of 0 VFDs was lower for patients treated by physiotherapists compared to those who were not [odds ratio (OR) = 0.36, 95% CI: 0.18–0.71]. Survival at 90 days was 96.0% in the physiotherapy group and 70.6% in patients not performing physiotherapy [hazard ratio (HR) = 0.14, 95% CI: 0.03–0.71]. Number of VFDs was not associated with body mass index (BMI), sex, or P/F at ICU admission for individuals with at least 1 day off the ventilator.

**Conclusion:**

In patients with COVID-19 admitted to ICU during the first pandemic wave and treated by physiotherapists, the number of days alive and free from MV was lower compared to patients who did not perform respiratory physiotherapy. Survival at 90 days in the physiotherapy group was greater compared to no physiotherapy. These findings may be the starting point for further investigation in this setting.

## Introduction

Early physical and occupational therapy is safe and well-tolerated in mechanically ventilated critically ill patients, and results in better functional outcomes at hospital discharge (1). Early mobilization in the Intensive Care Unit (ICU) was shown to decrease the duration of mechanical ventilation (MV) and shortening the length of ICU stay (2). In patients with acute respiratory distress syndrome (ARDS), early rehabilitation is associated with a reduction of the functional impairment due to the prolonged duration of ventilatory support and ICU stay (3). However, conflicting results were reported on the impact of active mobilization and rehabilitation in ICU on survival (4).

Physiotherapy and rehabilitation interventions have been recommended to minimize the functional sequelae of Coronavirus Disease (COVID-19) in mechanically ventilated patients admitted to ICU (5–8). Notably, critically ill patients with COVID-19 associated ARDS (CARDS) may require longer rehabilitation time than patients with non-COVID ARDS (9, 10). Respiratory physiotherapy combines early mobilization and other techniques optimizing secretion clearance, gas exchange, lung recruitment, and aiding with weaning from MV. Recent evidence suggests that respiratory physiotherapy in mechanically ventilated patients with CARDS is safe and feasible, although the benefits of early rehabilitation in these patients remain unclear (11, 12).

The aim of the present study was to assess whether the number of ventilator-free days (VFDs) and alive at day 28 was different in mechanically ventilated patients with severe COVID-19 and admitted to ICU, who performed or not respiratory physiotherapy.

## Methods

### Study design

We planned a retrospective, pragmatic, observational study of consecutive mechanically ventilated patients with COVID-19 admitted to the ICU of four Italian referral centers. A pragmatic design allowed for relaxed inclusion criteria thus contributing to our understanding of the real-life implementation of respiratory physiotherapy in ICU (13). The study was approved by the Ethics Committee of the Fondazione IRCCS Ca' Granda Ospedale Maggiore Policlinico (Comitato Etico Milano Area 2, approval n. 966_2020bis) and subsequently by the local Ethics Committees of the participating sites. Informed consent was waived due to the retrospective nature of the study. Baseline characteristics, comorbidities, pharmacological therapies, and clinical outcomes of all patients were extracted from electronic health records, collected, and managed using an electronic database (REDCap) (14) hosted at the coordinating center in Milano. The trial protocol and outcomes measures were published before study initiation (NCT 05067907). This observational study is presented in accordance with the STROBE guidelines (15).

### Study population

Patients admitted to an ICU from March 1^st^, 2020 to May 30^th^, 2020 at four centers [Fondazione IRCCS Ca' Granda Ospedale Maggiore Policlinico (Milano), ASST Grande Ospedale Metropolitano Niguarda (Milano), Ospedale Policlinico San Martino (Genova), and APSS Provincia Autonoma di Trento Ospedale Santa Chiara (Trento)] were included in the study. Centers were selected among the hospitals of the northern regions of Italy, which were mainly hit by the first pandemic wave, according to the following criteria: (1) the presence of a dedicated COVID-19 ICU; and (2) an established physiotherapy team with experience in the cardio-respiratory field and in the care of critically ill patients. Patients were eligible once neuromuscular blocking agents (NMBAs) were withdrawn. At this time, each ICU team could assign patients to respiratory physiotherapy pragmatically, consistent with the clinical practice of each ICU team (16). Thus, it was possible to evaluate a control group of patients who did not receive physiotherapy. We analyzed patients aged ≥18 and <80 years, with a confirmed severe acute respiratory syndrome coronavirus 2 (SARS-CoV-2) infection, who developed ARDS (17) and required invasive MV. Previous cognitive impairment (i.e., mini-mental state examination <20) was considered an exclusion criterion.

### Standard of care procedures

All the clinical interventions, such as use of antibiotics, ventilatory strategy, laboratory testing, and hemodynamic management were left at the discretion of each ICU team and in accordance with the most updated guidelines at the time of the study (18).

### Respiratory physiotherapy intervention

In Italy, physiotherapists oversee both musculoskeletal and respiratory rehabilitation. Each study center was encouraged to follow the best practice guidelines (19–21) and their institutional protocols for the care of individuals with COVID-19. Respiratory physiotherapy treatments were administered 7 days per week. Interventions included early mobilization and respiratory care, as previously described (11, 12). All patients that received respiratory physiotherapy treatments were assessed by physiotherapists once free from NMBAs. The respiratory physiotherapy protocol included different levels of activities; when patients were partially sedated, intubated, and mechanically ventilated, the treatment mainly focused on respiratory and peripheral muscles training, reaching, and maintaining lateral, sitting, and vertical positions, passive and active mobilization in bed and activity of daily life (ADL) training to promote independence and bed mobility (1). If necessary, modification of MV settings during mobilization/exercise were adopted according to the respiratory need of the patient. Moreover, airway clearance strategies were proposed as well as lung expansion techniques (i.e., deep breathing exercises) (22). When patients were completely awake and started weaning from MV, physiotherapists assessed their ability to perform bed mobility activities and to reach and maintain sitting and vertical position while receiving minimal or no ventilator support. In this phase, physiotherapists assisted MV weaning, eventually supporting patient with non-invasive ventilation after extubation. Active and strengthening exercises, ADL training, and endurance training were performed. In patients with tracheostomy, physiotherapists evaluated cough efficacy, swallowing, and humidification need and the treatment included spontaneous breathing trials, tracheal suctioning as well as promoting early speech recovery.

### Outcomes

The primary outcome was VFDs, defined as the number of days alive and ventilator-free for at least 24 consecutive hours, and calculated as follows: VFDs = 0 if patients died within 28 days or if patients were still ventilated within or after 28 days; VFDs = 28 – x if patients were liberated from ventilation x days after withdrawal of NMBAs. Pre-specified secondary outcomes were the following: ICU length of stay, arterial partial pressure of oxygen (PaO_2_)/fraction of inspired oxygen (FiO_2_) (P/F) ratio at ICU discharge, ICU and hospital survival, and survival at 90-days after NMBAs withdrawal.

### Statistical analysis

Due to overwhelming workload during the pandemic, the original sample size of 340 patients based on a difference of two VFDs between groups decreased to 244 (two-sided alpha level of 0.01 and power 90%) because one center declined to participate after protocol approval. All descriptive statistics were reported as counts (percentage), median [interquartile range (IQR)], or mean [Standard Deviation (SD)]. Data analysis was conducted following recommended methodological standards (23, 24). To estimate an association between VFDs and respiratory physiotherapy that is unbiased by pretreatment group differences on other observed variables, we included the following covariates in a propensity score from a multivariable logistic regression model of respiratory physiotherapy: age, body mass index (BMI), hospital center, sex, number of days under NMBAs, occurrence of reintubation and pronation, and presence of tracheostomy. In the propensity score calculation, variables associated with the intervention and outcome were chosen for inclusion according to Brookhart et al. (25). The overlap propensity score weighting (OPSW) method was then applied, in which each patient's weight is the probability of that patient being assigned to the opposite intervention group (26). The OPSW mimics the characteristics of highly inclusive trial, and it is proven to optimize precision of the estimates (27). Balance was demonstrated by reporting the standardized difference in means (or percentage) between the group that received physiotherapy intervention and the group that did not receive physiotherapy intervention. A zero-inflated negative binomial model (ZINB) (28) was used to estimate the association of respiratory physiotherapy with the primary outcome, combining a negative binomial and a logit model. The effect size was estimated as incidence rate ratio (IRR) and odds ratio (OR), respectively, with their 95% confidence interval (CI), obtained using robust standard errors. The count part of the model (i.e., the negative binomial) was adjusted with age, BMI, sex, and P/F ratio at ICU admission whereas the inflation part (i.e., the logit model) used presence of tracheostomy and exposure to pronation as adjusting covariates. We further adjusted the count component with hospital centers interacting with physiotherapy. To estimate secondary outcomes, mean difference was used to describe P/F ratio at ICU discharge between groups. We used adjusted Cox proportional hazard regression models for survival endpoints: to increase statistical efficiency, analyses were further adjusted for age, sex, and P/F ratio at ICU admission, with physiotherapy interacting with hospital center strata; hazard ratios (HRs) were then calculated. Crude and weighted estimates are presented before and after OPSW along with 95% CI. All analyses were performed using the R software version 4.0.3 (29), with packages *cobalt* (30) and *pscl* (31) added.

## Results

**Figure 1:**
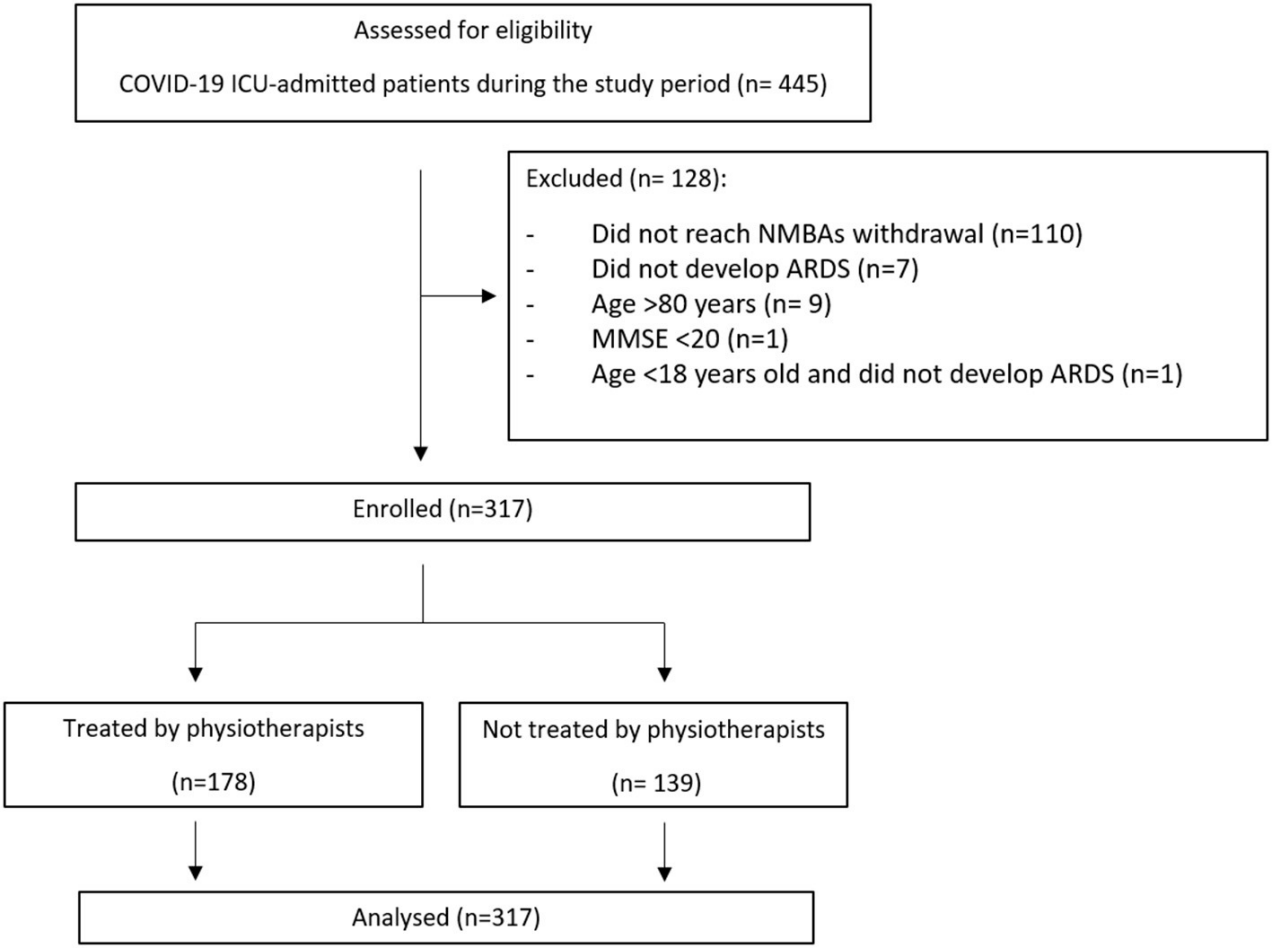
STROBE flow diagram. COVID-19, Coronavirus Disease 2019; ICU, Intensive Care Unit; NMBAs, neuromuscular blocking agents; ARDS, acute respiratory distress syndrome; MMSE, mini-mental state examination.

From March 1^st^ to May 30^th^, 2020, 317 consecutive patients [mean (SD) age 60.5(11.5) years and 73(25%) women] were enrolled (Figure 1). Once NMBAs were withdrawn, 178 individuals received respiratory physiotherapy and 139 did not. Characteristics of included patients are summarized in Table 1. Pharmacological treatments are reported in Supplementary Table 1. Patients had a substantial prevalence of hypertension, without any remarkable difference between patients treated or not by physiotherapists (Table 2). Respiratory physiotherapy interventions are reported in Table 3. The most common respiratory physiotherapy maneuvers were active mobilization (89.6% of patients), reaching sitting at the edge of bed position (82.1% of patients) and muscle strength exercises (79.8% of patients); respiratory care was performed as well in 83.7% of patients with airways clearance techniques to decrease airways resistance and 87.5% of patients with prone or lateral positioning to improve gas exchange.

**Table 1:** Baseline characteristics of patients with COVID-19 before and after overlap propensity score-weighting.

	**Pre-weighting**			**Post-weighting**		
	**No physiotherapy** **(*n* = 139)**	**Physiotherapy** **(*n* = 178)**	**SMD**	**No physiotherapy** **(*n* = 139)**	**Physiotherapy** **(*n* = 178)**	**SMD**
**Age, years**	61.3 (13.1)	59.9 (10.1)	0.122	60.3 (13.2)	60.3 (10.1)	<0.001
**Sex**						
F	38 (27.3)	41 (23.0)	0.099	24.9	24.9	<0.001
M	101 (72.7)	137 (77.0)		75.1	75.1	
**BMI, kg/m** ^**2**^	28.3 (5.2)	28.2 (4.7)	0.023	28.2 (5.2)	28.2 (5.0)	<0.001
**Ethnicity**, ***n***						
Caucasian (European)	117 (84.2)	164 (92.1)	0.345	84.4	91.4	0.295
Other	22 (15.8)	14 (7.9)		15.8	8.6	
**PaO**_**2**_**/FIO**_**2**_ **ratio**	145.9 (60.0)	133.4 (53.1)	0.219	141.9 (58.8)	141.1 (58.2)	0.014
**Tracheostomy**, ***n***						
No	126 (90.6)	151 (84.8)	0.178	88.3	88.3	<0.001
Yes	13 (9.4)	27 (15.2)		11.7	11.7	
**Pronation**, ***n***						
No	93 (66.9)	99 (55.6)	0.233	63.8	63.8	<0.001
Yes	46 (33.1)	79 (44.4)		36.2	36.2	
**ECMO**, ***n***						
No	138 (99.3)	177 (99.4)	0.020	98.8	99.2	0.037
Yes	1 (0.7)	1 (0.6)		1.2	0.8	
**CRRT**, ***n***						
No	134 (96.4)	167 (93.8)	0.120	95.1	96.1	0.047
Yes	5 (3.6)	11 (6.2)		4.9	3.9	
**Reintubation**, ***n***						
No	133 (95.7)	173 (97.2)	0.081	96.3	96.3	<0.001
Yes	6 (4.3)	5 (2.8)		3.7	3.7	
**NMBAs, days**	8.3 (9.0)	12.8 (12.3)	0.416	9.8 (10.6)	9.8 (9.1)	<0.001

Data are reported as mean (SD) or count (percentage) if not otherwise specified. Reported is either the OPSW mean (SD) or proportion. SMD, standardized mean or percentage difference; BMI, body mass index; ECMO, extra corporeal membrane oxygenation; CRRT, continuous renal replacement therapy; NMBAs, neuromuscular blocking agents.

**Table 2:** Comorbidities (%).

	**Pre-weighting**		**Post-weighting**		***P*-value^a^**
	**No physiotherapy (*n* = 139)**	**Physiotherapy (*n* = 178)**	**No physiotherapy (*n* = 139)**	**Physiotherapy (*n* = 178)**	
Cardiovascular diseases	5.8	8.4	6.5	6.1	0.392
Hypertension	44.8	42.8	43.3	47.5	0.469
Diabetes	16.2	14.5	16.6	15.6	0.519
Dyslipidemia	1.4	2.2	2.4	1.3	0.699
Chronic Respiratory diseases	9.4	16.9	10.5	15.3	0.068
Psychiatric disorders	2.2	1.7	2.9	0.7	1.000
*Ictus cerebri*	2.9	1.9	4.7	3.5	0.405
Neuropathy	1	–	0.8	–	0.493
Neoplastic disorders	3.6	2.8	4.6	3.3	0.753
Others	5	8.4	7	5.9	0.272

Reported is either raw or OPSW % proportion.

^*a*^Post-weighting Fisher's exact test.

**Table 3:** Respiratory physiotherapy intervention (*n* = 178).

**Activities**	**Pre-weighting**	**Post-weighting**
**Early mobilization**		
Passive mobilization	31	40.8
Active mobilization	93.7	89.6
Muscle strength exercises	83.9	79.8
Promote autonomy with ADL	81	73.9
Sitting at the edge of bed	76.4	82.1
Verticalization	41.4	48.8
Ambulation	24.1	36.4
**Respiratory care**		
Positioning to increase ventilation/perfusion ratio	93.2	87.5
Airways clearance	82.4	83.7
Lung expansion exercises	59.7	64.3
Management of inhaled therapy	5.1	6.8
Management of oxygen therapy	52.8	68.6
NIV/CPAP management	47.2	52.3
Management of tracheostomy and cannula weaning	48.9	46.2
Support to IMV weaning	72.7	67.2

Reported is either raw or OPSW % proportion. ADL, activity of daily life; NIV, non-invasive ventilation; CPAP, continuous positive airway pressure; IMV, invasive mechanical ventilation.

### Primary outcome

**Figure 2:**
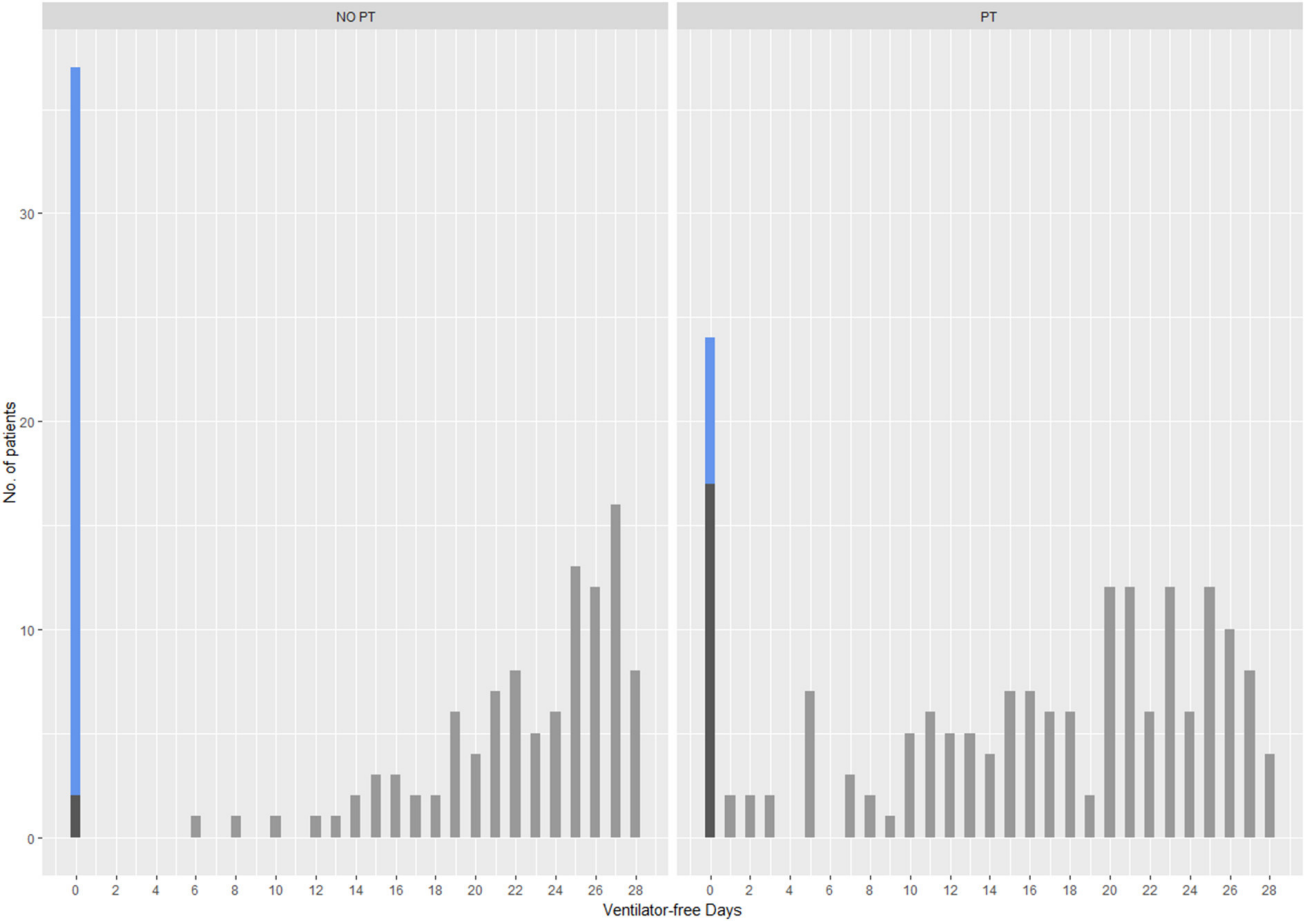
Distributions of VFDs between patients treated (PT) or not (NO-PT) by physiotherapists at 28 days. Blue bars denote patients who died (VFD = 0), dark gray bars denote patients alive with VFD = 0 and light gray bars identify patients alive with VFDs > 0.

At day 28, 178 patients performing respiratory physiotherapy had a median of 18 ventilator-free days (IQR, 10; 24), and 139 patients not followed by physiotherapists had a median of 21 VFDs (IQR, 0; 26). Distribution of VFDs is depicted in Figure 2. Patients referred to respiratory physiotherapy were less likely to have 0 VFDs compared to those not treated (OR 0.36, 95%CI: 0.18; 0.71). The ZINB estimates are reported in Supplementary Table 2. From the inflation part, presence of tracheostomy increased the odds of having 0 VFDs by 2.1, but with a high uncertainty (95%CI: 0.92; 4.89). Patients treated with prone position were more likely to have 0 VFDs (OR 2.1, 95%CI: 1.09; 4.23). The count part shows that average number of VFDs was lower in the respiratory physiotherapy group compared to the other group (IRR 0.86, 95%CI: 0.78; 0.95). Older patients have slightly fewer VFDs (IRR 0.99, 95%CI: 0.99; 1.0). No evidence of statistical association was found between VFDs and BMI (IRR 1.0; 95%CI: 0.99; 1.01), sex (IRR 0.97, 95%CI: 0.89; 1.05), and P/F ratio at ICU admission (IRR 1.0, 95%CI: 1.0; 1.0). The number of VFDs was not different across centers whether patients received or not physiotherapy (*P* = 0.962); however, hospital centers did account for statistically significant differences in the VFDs (*P* < 0.001) (Table S2).

### Secondary outcomes

The mean P/F ratio at ICU discharge was 35.4 points higher in patients receiving respiratory physiotherapy than those who did not perform respiratory physiotherapy (95%CI: 12.6; 58.3 points). Patients receiving respiratory physiotherapy had 8.2 days longer mean ICU stay than those who did not perform respiratory physiotherapy (95%CI: 5; 11.4 days), indicating a narrow range of plausible true length of stay (Table 4). Among patients treated by physiotherapists, 96% (vs. 70.6% in the group not performing respiratory physiotherapy) survived up to 90 days (HR 0.14; 95%CI: 0.03; 0.71). No interaction was found between respiratory physiotherapy and centers in 90-day survival (*P* = 0.7018) as well as no evidence of association between respiratory physiotherapy and ICU survival (HR: 0.22; 95%CI: 0.04; 1.20). Overall, 96% of patients treated by physiotherapists, compared to 70% of patients in the group without physiotherapy was discharged alive from hospital (*P* < 0.001).

**Table 4:** Outcomes in patients hospitalized with COVID-19 before and after overlap propensity score-weighting.

	**Pre-weighting**			**Post-weighting**		
	**No physiotherapy** **(*n* = 139)**	**Physiotherapy** **(*n* = 178)**	**Estimate** **(95%CI)**	**No physiotherapy** **(*n* = 139)**	**Physiotherapy** **(*n* = 178)**	**Estimate** **(95%CI)**
**Primary outcome**
Days alive and ventilator-free at 28 days						
Median (IQR)	21 (0; 26)	18 (10, 23)	OR 0.35 (0.19; 0.63) IRR 0.83 (0.71; 0.97)^a^	21 (0; 26)	18 (10, 24)	OR 0.36 (0.18; 0.71) IRR 0.86 (0.78; 0.95)^a^
28-days survival						
*n* (%)	104 (74.8)	171 (96.1)		71	96	
Days free of mechanical ventilation among survivors						
Median (IQR)	24 (20, 26)	18 (11, 23)		24 (20, 27)	20 (11, 25)	
**Secondary outcomes**
ICU length of stay						
Mean (SD)	17.7 (11.9)	28.8 (16.6)	11.1 (7.83; 14.4)^b^	18.4 (12.7)	26.6 (16.1)	8.2 (5; 11.4)^b^
PaO_2_/FIO_2_ ratio at ICU discharge						
Mean (SD)	237.9 (103.1)	277.2 (98.5)	39.4 (17; 61.7)^b^	234.2 (106.9)	269.7 (99.6)	35.4 (12.6; 58.3)^b^
**ICU survival**						
*n* (%)	108 (77.7)	170 (95.5)		73.9	96	
Median time (IQR)	42.0 (23.0; –)	94.0 (–; –)	0.13 (0.02; 0.97)^c^	35.0 (23.0; 52.0)	94.0 (–; –)	0.22 (0.04; 1.20)^c^
**90-day survival**						
*n* (%)	103 (74.1)	170 (95.5)		70.6	96	
Median time (IQR)	– (52.0; –)	– (–; –)	0.18 (0.04; 0.81)^c^	– (42.0; –)	– (–; –)	0.14 (0.03; 0.71)^c^

ICU, Intensive Care Unit; SD, standard deviation; IQR, interquartile range; PaO_2_, arterial partial pressure of oxygen; FiO_2_, fraction of inspired oxygen.

^*a*^ Estimates are odds ratio (OR) from adjusted Zero Inflated Negative Binomial Model (inflation part) and incidence rate ratio (IRR) form the count part with 95% confidence interval (CI).

^*b*^ Estimates are mean difference from linear regression.

^*c*^ Estimates are adjusted hazard ratio from Cox proportional model.

## Discussion

In this pragmatic, observational study of critically ill patients with COVID-19, we found that respiratory physiotherapy after NMBAs withdrawal was associated with less VFDs compared to no physiotherapy. In addition, patients receiving respiratory physiotherapy showed increased P/F ratio at ICU discharge, prolonged ICU stay duration; most of the patients were discharged alive from hospital. Probability of zero VFDs was lower in patients receiving respiratory physiotherapy. There was no evidence of association between VFDs and BMI, sex, or P/F ratio at ICU admission.

In the present study we included many patients treated by physiotherapists in four different ICUs, making our findings rather generalizable. The multi-modal intervention of physiotherapists in ICU during the first wave of COVID-19 pandemic, represents a pragmatic scenario of the current clinical practice (13). The primary outcome was VFDs because it is a clinically relevant measure in ICU, likely affected by both early mobilization (32) and respiratory care. In contrast to our original hypothesis, we observed that patients who received respiratory physiotherapy had less VFDs than patients who did not. Several factors may explain this result. Liberation from MV was defined for a period ≥24 h, implying that interval disconnections were not considered (this is especially true for patients with tracheostomy in whom count of VFDs started after the last successful disconnection from the ventilator). Different features could have possibly influenced the respiratory physiotherapy pathway. Firstly, *delirium* can affect patients' cooperation and their ability to reach functional goals (33); secondly, illness severity and comorbidities, identified by severity scores at ICU admission, may be responsible for difficult weaning, thus requiring more frequent and demanding interventions (33, 34). Thirdly, respiratory physiotherapy uses different methods or techniques of treatment (35), that allow to personalize each intervention, tuning frequency, timing, and dosage according to patient's needs and available evidence. The heterogeneity of ICU-related respiratory physiotherapy interventions described in the literature makes it difficult to measure the effectiveness of a specific treatment (36). To date, no solid short-term outcomes exist to support one respiratory physiotherapy technique over another in the ICU setting, especially if we consider the unprecedented pandemic scenario in which all healthcare professions worked with limited evidence-based guidance. We might speculate that VFDs may not be an appropriate endpoint to evaluate respiratory physiotherapy in mechanically ventilated patients. A recent systematic review (32) reports only two studies over six with a net effect of early mobilization on VFDs with high heterogeneity, thus suggesting the small responsiveness of VFDs to respiratory physiotherapy interventions. The fundamental question remains if respiratory physiotherapy has a combined and consistent effect on both mortality and on MV duration, and if this can be captured by the composite VFDs outcome. Thus, the use of time to extubation may be more appropriate since it is associated with both ICU- and post-discharge morbidity and mortality in critically ill adults, representing a patient-centered outcome measure (37). Other outcome measures might include re-intubation rate, which is potentially associated with acquisition of ventilatory acquired pneumonia (22).

The number of VFDs differed among centers likely due to regional disparities in the spread of COVID-19 with hospitals facing an extreme burden and very challenging work conditions. We hypothesize that the decision to address patients to physiotherapists varied among centers. However, there was no association between respiratory physiotherapy among centers and VFDs, which indirectly means that different physiotherapists used similar clinical approaches. This is further supported by the lack of interaction of physiotherapy with center in the survival model.

Patients treated by physiotherapists showed a longer duration of MV and ICU stay but better oxygenation at ICU discharge and 90-day survival. The improvement in oxygenation after respiratory physiotherapy has been previously reported (38). Survival rate is not in line with previous studies (39) since those patients who did not reach the NMBAs withdrawal and therefore died in ICU were not eligible. The COVID-19 pandemic forced the medical team to allocate treatments in resource-limited circumstances (40), including respiratory physiotherapy (41). In our study, each ICU medical team decided autonomously if patients needed or not respiratory physiotherapy and this might have introduced a selection bias. As a matter of fact, indications for respiratory physiotherapy initiation are currently missing. We addressed this issue in the study design by enrolling only patients who achieved the NMBAs withdrawal, which is the moment when respiratory physiotherapy can potentially start. To account for the selection bias, we used the OPSW. Therefore, presented estimates are measures of association between respiratory physiotherapy/no-respiratory physiotherapy and VFDs, with respect to a population of patients at equipoise either to receive treatment or not (27). Nevertheless, we cannot exclude that a further bias may had occurred in the group of individuals deemed more likely to survive, resulting in respiratory physiotherapy requested for individuals who were experiencing difficult weaning, leaving others exposed to natural disease recovery. All these implicit decisions could be plausibly responsible for the survival benefit as seen in the respiratory physiotherapy group but could also account for the longer MV duration and ICU stay. Considering that respiratory physiotherapy interventions in ICU are not limited to early mobilization but also to respiratory care (42), especially in patients with respiratory diseases, yet it remains crucial to understand how intensivists decide which patient needs or not these treatments. Where physiotherapists are not part of the ICU staff or not involved in the routine assessment of individuals hospitalized in ICU (16, 43, 44), goal-oriented allocation criteria shared between physiotherapists and intensivists are needed. Under this perspective, we convene that respiratory physiotherapy initiation in ICU is not a random process. Decisional rules should be further explored and validated in terms of disease-oriented and patient-important outcomes. Patient assessment and proactive respiratory physiotherapy remain crucial to optimize treatments and ICU staff utilization.

### Limitations

Several limitations must be acknowledged. First, this is an observational retrospective study which can be susceptible to selection bias. Second, unmeasured confounders might have been overlooked since different ventilatory strategies among centers could have played a role in the MV duration. Third, despite respiratory physiotherapy treatments were individualized, the dose, intensity, and frequency of each intervention were not evaluated. Finally, we cannot exclude that our results could be further biased by regional differences in COVID-19 management and hospital staff workload; however, we used center as adjusting factor (37).

## Conclusion

In mechanically ventilated COVID-19 patients admitted to ICU, respiratory physiotherapy intervention, including early mobilization and respiratory care, was associated with a lower number of VFDs compared to patients who were not treated by physiotherapists. Future studies on respiratory physiotherapy in ICU should be designed considering responsive outcomes to both early mobilization and respiratory care, given that physiotherapy is an ensemble of methods and techniques with different rationales.

## Data availability statement

The raw data supporting the conclusions of this article will be made available by the authors, without undue reservation.

## Ethics statement

The studies involving human participants were reviewed and approved by Ethics Committee of the Fondazione IRCCS Ca' Granda Ospedale Maggiore Policlinico (Comitato Etico Milano Area 2, approval n. 966_2020bis). Written informed consent for participation was not required for this study in accordance with the national legislation and the institutional requirements.

## Author contributions

EP: conceptualization, resources, methodology, supervision, and project administration. SG: methodology, formal analysis, visualization, data curation, writing original draft, writing review, and editing. VR and FB: conceptualization, methodology, investigation, data curation, writing review, and editing. MS: conceptualization, methodology, data curation, investigation, data curation, writing original draft, writing review, and editing. DT and SC: investigation and data curation. VT and CC: investigation, resources, writing review, and editing. DB: investigation, writing review, and editing. MP: conceptualization, methodology, writing original draft, writing review, and editing. RF and PP: resources, writing review, and editing. GG: resources, supervision, writing review, and editing.

## Funding

This study was (partially) funded by Italian Ministry of Health—Current research IRCCS.

## Conflict of interest

The authors declare that the research was conducted in the absence of any commercial or financial relationships that could be construed as a potential conflict of interest.

## Publisher's note

All claims expressed in this article are solely those of the authors and do not necessarily represent those of their affiliated organizations, or those of the publisher, the editors and the reviewers. Any product that may be evaluated in this article, or claim that may be made by its manufacturer, is not guaranteed or endorsed by the publisher.
