# Supplementary material for: Association of ventilator-free days with respiratory physiotherapy in critically ill patients with Coronavirus Disease 2019 (COVID-19) during the first pandemic wave. A propensity score-weighted analysis
Source: Front Med (Lausanne). 2022 Sep 12;9:994900. doi: 10.3389/fmed.2022.994900 (PMC9510617; doi:10.3389/fmed.2022.994900)
Supplement: Supplementary file 1 [file Table_1.DOCX]

**SUPPLEMENTARY**

| **Tab. S1 - Drugs (%)** | | | | | |
| --- | --- | --- | --- | --- | --- |
|  |  | **Pre-weighting** |  | **Post-weighting** |  |
|  |  | **No Physiotherapy (n=139)** | **Physiotherapy (n=178)** | **No Physiotherapy (n=139)** | **Physiotherapy (n=178)** |
| **Corticosteroids^a^** | |  |  |  |  |
|  | Yes | 41 | 42.1 | 46.6 | 37.4 |
|  | No | 13.7 | 37.1 | 18.5 | 27.3 |
|  | Not Known | 45.3 | 20.8 | 34.9 | 35.3 |
| **Inhaled bronchodilators^b^** | |  |  |  |  |
|  | Yes | 3.6 | 14.6 | 6.3 | 9.1 |
|  | No | 51.8 | 64.6 | 59.4 | 55.6 |
|  | Not Known | 44.6 | 20.8 | 34.3 | 35.3 |
| **Sedatives^c^** | |  |  |  |  |
|  | Yes | 45.3 | 72.5 | 55 | 54.4 |
|  | No | 10.1 | 7.3 | 10.7 | 11.4 |
|  | Not Known | 44.6 | 20.2 | 34.3 | 34.2 |
| *Reported is either raw and OPSW %proportion; Post-matching Chi-square test: ^a^ P=0.199, ^b^ P=0.690, ^c^ P=0.986* | | | | |  |

| **Table S2. Unadjusted and adjusted VFDs after overlap propensity score-weighting** | | | |
| --- | --- | --- | --- |
| ***Models*** |  | ***Covariates*** | ***Days alive and ventilator free at 28 days*^1^** |
| Unadjusted weighted ZINB | count | *PT Yes versus PT No* | 0.82 (0.76; 0.89), P<0.001 |
|  | inflation | *PT Yes versus PT No* | 0.37 (0.19; 0.71), P=0.003 |
|  |  |  |  |
| Adjusted weighted ZINB^a^ | count | (Intercept) | 23.73 (21.63; 26.03) |
|  |  | *PT Yes versus PT No* | **0.86 (0.78; 0.95), P=0.003** |
|  |  | *Age* | **0.99 (0.99; 1.00), P<0.001** |
|  |  | *BMI* | 1.00 (0.99; 1.01), P=0.915 |
|  |  | *Center B versus Center A* | **1.14 (1.02; 1.27), P=0.024** |
|  |  | *Center C versus Center A* | **0.84 (0.75; 0.93), P=0.001** |
|  |  | *Center D versus Center A* | **0.75 (0.67; 0.83), P<0.001** |
|  |  | *PT x Center B* | 1.00 (0.86; 1.17), P=0.971 |
|  |  | *PT x Center C* | 0.94 (0.76; 1.15), P=0.537 |
|  |  | *PT x Center D* | 0.96 (0.80; 1.15), P=0.673 |
|  |  | *Males versus females* | 0.97 (0.89; 1.05), P=0.439 |
|  |  | *P/F ratio at ICU admission* | 1.00 (1.00; 1.00), P=0.118 |
|  | inflation | (Intercept) | 0.29 (0.17; 0.47) |
|  |  | *PT Yes versus PT No* | **0.36 (0.18; 0.71), P=0.003** |
|  |  | *Tracheostomy versus No tracheostomy* | 2.12 (0.92; 4.89), P=0.076 |
|  |  | *Pronation versus ever-pronated* | **2.15 (1.09; 4.23), P=0.028** |
| ^1^Estimates are odds ratio (inflation part) and incidence rate ratio (count part) from Zero Inflated Negative Binomial Models (ZINB) with robust standard error and 95% Confidence Interval;  ^a^Adjusted weighted ZINB is superior to unadjusted weighted ZINB according to Vuong statistic (-4.60, P<0.001). Abbreviations: VFDs = Ventilator Free Days; PT = Physiotherapy; BMI = Body Mass Index; ICU = Intensive Care Unit; P/F = arterial partial pressure of oxygen (PaO_2_)/fraction of inspired oxygen (FiO_2_) ratio | | | |
